# Studies on a widely-recognized snail model species (*Lymnaea stagnalis*) provide further evidence that vertebrate steroids do not have a hormonal role in the reproduction of mollusks

**DOI:** 10.3389/fendo.2022.981564

**Published:** 2022-09-08

**Authors:** István Fodor, Tamar Schwarz, Bence Kiss, Antal Tapodi, János Schmidt, Alex R. O. Cousins, Ioanna Katsiadaki, Alexander P. Scott, Zsolt Pirger

**Affiliations:** ^1^ Ecophysiological and Environmental Toxicological Research Group, Balaton Limnological Research Institute, Eötvös Loránd Research Network (ELKH), Tihany, Hungary; ^2^ Centre for Environment, Fisheries and Aquaculture Research, Weymouth Laboratory, Weymouth, United Kingdom; ^3^ Institute of Biochemistry and Medical Chemistry, Medical School, University of Pécs, Pécs, Hungary; ^4^ Lowestoft Laboratory, Centre for Environment, Fisheries and Aquaculture Science, Lowestoft, United Kingdom

**Keywords:** mollusk, *Lymnaea stagnalis*, neuroendocrine, sex steroid uptake and metabolism, immunohistochemistry, western blot, mass spectrometry

## Abstract

Experiments were carried out to determine whether, as with other mollusks that have been studied, the snail, *Lymnaea stagnalis*, can absorb, esterify and store vertebrate steroids that are present in the water. We also carried out experiments to determine whether neural tissues of the snail could be immunohistochemically stained with an antibody to human aromatase (a key enzyme that catalyzes the conversion of testosterone [T] to 17β-estradiol [E_2_]); and, if so, to determine the significance of such staining. Previous studies on other mollusks have reported such staining and have proposed this as decisive evidence that mollusks have the same steroid synthesis pathway as vertebrates. We found that snails absorb, esterify and retain esterified T, E_2_, progesterone and ethinyl-estradiol (albeit with an absorption rate about four times slower, on a weight basis, than the mussel, *Mytilus edulis*). We also found that not only anti-human aromatase, but also anti-human nuclear progesterone receptor (nPR) and anti-human gonadotropin-releasing hormone antibodies immunohistochemically stained snail neural cells. However, further experiments, involving gel electrophoretic separation, followed by immunostaining, of proteins extracted from the neural tissue, found at least two positively-stained bands for each antibody, none of which had masses matching the human proteins to which the antibodies had been raised. The anti-aromatase antibody even stained the 140 kDA ladder protein used as a molecular weight marker on the gels. Mass spectrometric analysis of the bands did not find any peptide sequences that corresponded to the human proteins. Our findings confirm that the presence of vertebrate-like sex steroids in molluscan tissues is not necessarily evidence of endogenous origin. The results also show that immunohistochemical studies using antibodies against human proteins are grossly non-specific and likely to have little or no value in studying steroid synthesis or activity in mollusks. Our conclusions are consistent with the fact that genes for aromatase and nPR have not been found in the genome of the snail or of any other mollusk. Our overarching conclusion, from this and our previous studies, is that the endocrinology of mollusks is not the same as that of humans or any other vertebrates and that continuing to carry out physiological and ecotoxicological studies on mollusks on the basis of this false assumption, is an unconscionable waste of resources.

## Introduction

Ever since the discovery of the major vertebrate sex steroids, progesterone (P), testosterone (T), and 17β-estradiol (E_2_), in mollusks in the late 1950s, a large body of literature has attempted to provide evidence of their role as hormones in mollusks as in vertebrates [reviewed by (1–3)]. A common line of evidence is that sex steroids are present (and, indeed, easy to measure) in tissues of a wide range of mollusks and that in some (but, it must be stressed, not all) studies, their concentrations sometimes show an association with stages of gonad development, sometimes show differences between tissues and sexes, and sometimes appear to be altered in the presence of contaminants [reviewed by (2, 4–7)]. Another line of evidence is that cells in mollusk tissues can be immunohistochemically stained with antibodies raised against mammalian proteins that are associated with sex steroid synthesis (e.g. Cytochrome P450 [CYP] 11A, CYP19A, and 17β-hydroxysteroid dehydrogenase); or steroid reception (e.g. nuclear estrogen receptor [nER]) (8–14).

In regard to the first line of evidence, it has been established, overwhelmingly, since 2000 that mollusks can readily absorb P, T, and E_2_ from the environment and store them and/or their metabolites for weeks to months in the form of fatty acid esters (6, 15–29). Also, ethinyl-estradiol (EE_2_; which is the estrogenic component of “The Pill”) has been detected in the flesh of wild-caught mollusks (30–33). It is highly improbable that the animals would have made this compound themselves as it is purely a man-made chemical. In view of such findings, and the fact that vertebrate steroids are widespread in the environment, it is legitimate to ask how much (if any at all) of the vertebrate sex steroids found in molluscan tissues are of endogenous origin.

Another problem concerning the putative role of vertebrates steroids in the mollusks is the fact that three crucial steps in the classical vertebrate steroid biosynthetic pathway - cholesterol side-chain cleavage, 17-hydroxylation, and aromatization – appear to be either absent or have very weak activity in mollusks (**Supplementary Figure 1**) [reviewed by (2, 3, 34–36)]. Most importantly, the protein homologues of the enzymes that catalyze the first and third of these reactions in vertebrates, CYP11A and CYP19A, respectively, as well as functional sex steroid nuclear receptors, have so far not been found in molluscan genomes (**Supplementary Figure 1** **;** **Supplementary Table 1**) [reviewed by (2, 3, 34–36)]. These facts are at odds, however, with the immunohistochemical evidence in the literature – which show that tissues in a range of mollusks can be stained with antibodies to human enzymes and steroid nuclear receptors.

We have recently investigated the neuronal transcriptome of the great pond snail (*Lymnaea stagnalis*) and confirmed the absence of several of the key protein sequences that would be required to accomplish full sex steroid biosynthesis and sex steroid receptor-mediation as found in vertebrates (**Supplementary Table 2**) (37). The aim of the present study was to provide further evidence for the notion that molluscan endocrinology differs from the well-characterized vertebrate endocrine system using *L. stagnalis*. This snail has been a widely used model organism in neuroscience, neuroendocrinology, and ecotoxicology for decades [reviewed by (38–48)], and is thus highly appropriate for such studies. To accomplish our aim, we exposed *L. stagnalis* to radiolabeled sex steroids (E_2_, T, P, and EE_2_) to determine if they are absorbed from water and subsequently depurated, and the rates at which these occur. Also, we performed immunohistochemistry (IHC) using commercially-available antisera generated against three mammalian proteins, CYP19A, nuclear progesterone receptor (nPR), and gonadotropin releasing hormone (GnRH), associated with vertebrate sex steroid synthesis and reception to investigate whether these yielded positive signals in the central nervous system (CNS). We also used Polyacrylamide Gel (PAGE) separation of CNS extracts, followed by Western blotting (WB), and mass spectrometry (MS) analyses to try and identify (or at least partially characterize) proteins that showed cross-reactivity with these antibodies.

## Materials and methods

### Chemicals

17β-[2,4,6,7,16,17-^3^H]- E_2_ ([^3^H]-E_2_), [1,2,6,7-^3^H]-T ([^3^H]-T), [1,2,6,7-^3^H]-P ([^3^H]-P), and 17-[6,7-^3^H(N)]- EE_2_ ([^3^H]-EE_2_), were purchased from American Radiolabeled Chemicals, Inc. (101 ARC Dr. St. Louis, MO 63146 USA).

### Experimental animals

For IHC, WB, and MS experiments, *L. stagnalis* specimens were obtained from the laboratory-bred stocks of the Balaton Limnological Research Institute (Hungary). Snails were kept in large holding tanks (with a 100 individuals/tank stocking density) containing 10L oxygenated artificial snail water with low copper content at a constant temperature of 20°C ( ± 1°C) on a light:dark regime of 12 h:12 h. Specimens were fed on lettuce *ad libitum* three times a week. All animals used in the experiment originated from the same breeding cohort and were thus all of the same age (five months old, mature snails). All procedures were performed according to the protocols approved by the Scientific Committee of Animal Experimentation of the Balaton Limnological Research Institute (VE-I-001/01890-10/2013).

The steroid uptake and depuration experiments were carried out at the Cefas, Weymouth Laboratory in the UK. *L. stagnalis* specimens were supplied by Blades Biological (Kent, UK). The husbandry of the snails used in the experiments was very similar to that described above. Briefly, *L. stagnalis* were housed in 100 L glass aquaria (stocking density 3.6 _[snails]_/L; culture media was dechlorinated ambient tap water) in controlled temperature room at 20 ± 1°C, and 16 hour light: dark photoperiod with light of natural wavelength (400-700nm) and an intensity of 400-500 Lux. Specimens were fed a diet of non-organic round lettuce *ad libitum*.

### Laboratory exposures of *L. stagnalis* to labeled steroids

The experiment was designed to investigate uptake, metabolism, and depuration of radioactive-labeled steroids by *L. stagnalis*. The methodology was based on previously published protocols (15–18) with slight modifications (e.g., freshwater instead of seawater, shorter incubation and depuration period) - the details are provided in **Table 1**. All procedures involved placing the animals in containers with freshwater (pre-aerated water), adding labelled steroids to the water using ethanol as a carrier (<0.01% final solvent concentration), and then taking 1 mL water samples (immediately mixed with 7 mL scintillation fluid) every two hours to measure the amount of radioactivity remaining in the water. In all cases, three sorption control vessels (radioactive steroid but no animals) were in place to determine how much radioactivity was being lost to sorption to the vessels. Scintillation counting was carried out with color quench correction on a Tricarb 2910 scintillation counter (PerkinElmer). For each steroid exposure, five animals (one from each replicate) were immediately frozen after exposure (day 0 of depuration) and the remaining five were placed in a fresh container with 4 L aerated freshwater for depuration under semi-static conditions. The animals were sampled and frozen on day 10.

**Table 1:** Experimental conditions and basic animal data.

Experiment	Water Vol. (L)	Number of animals	Replicates	Label conc. at first sampling point (µCi L^-1^)	Label conc. at first sampling point (ng L^-1^)	Exposure time (h)	Volume of water animal^-1^ (mL)	Mean tissue wet weight (g)
E_2_	0.3	2	5	2.6	5.9	8	150	0.96
EE_2_	0.3	2	5	2.7	17.2	8	150	1.00
T	0.3	2	5	3.5	7.1	8	150	1.16
P	0.3	2	5	2.7	9.1	8	150	1.03

### Calculation of clearance rates

The rates at which individual snails initially cleared steroids from water (i.e. clearance rates) were calculated for each steroid as follows. The percentage radiolabel remaining in the water from each treatment was first corrected for loss of radiolabel due to sorption. Label disappearance data were fitted to linear decay curves using Sigmaplot (Systat Software Inc, TW4 6JQ, London, UK.). The fitted curves were used to calculate the proportion of radiolabel that had been removed from the water between 0 and 1.5 h. This proportion (r) was used in the following equation to derive the ‘clearance rate’ of an individual snail (mL animal^-1^h^-1^) at the start of each exposure period:


$$Initial\;clearace\;rate=\frac{rV}{1.5n}$$

where r = proportion of radiolabel removed over the first 1.5 h; V= total volume of water in the container (mL); n = number of animals in the container.

### Steroid extraction and separation methods

For extraction of radioactive steroids from snail tissues, the animals sampled after the 8 h of exposure (i.e. day 0) and depuration (i.e. day 10) were defrosted at room temperature, shucked, blotted dry, and weighed. The tissues were extracted with methanol and ethyl acetate using Method 2 as described previously (15). All extracts had a final volume of 5 mL and were stored at −20°C.

To separate the free, water-soluble, and esterified metabolites, a portion of each extract (800 μL) was evaporated with nitrogen gas and partitioned using the separation method described before (15). Briefly, the dried extracts were shaken twice with a heptane/80% ethanol mix. The highly lipophilic steroid esters partitioned into the heptane phase. The 80% ethanol fraction contained the free (i.e. non-conjugated) and water-soluble metabolites. This was dried completely, reconstituted in water, and extracted with diethyl ether resulting in the separation of the water-soluble metabolites from the free steroids. Saponification of the heptane fraction to release the free steroids was also performed as described previously. A sample (500 μL) of each fraction was mixed with 7 mL scintillation fluid for counting.

### Immunohistochemistry

The whole CNS was dissected from individual snails (n=5). Tissue fixation, sectioning, and IHC procedure were implemented following a previously published protocol (49). Briefly, CNSs were pinned out on a Sylgard-coated dish containing 4% paraformaldehyde in 0.1 M phosphate buffer (pH=7.4) overnight at 4°C. After washing with phosphate-buffered saline (PBS; 137 mM NaCl, 10 mM Na_2_HPO_4_, 1.8 mM KH_2_PO_4_, pH=7.4), fixed tissues were cryoprotected in 20% glucose solution for 4 hours at room temperature and embedded into Cryomatrix (#6769006, Thermo Scientific). A series of 12-14 μm-thick cryostat sections were cut and thaw-mounted onto gelatin-aluminium-coated slides. In the case of the comparison of specific anti-*Lymnaea* GnRH/corazonin (ly-GnRH/CRZ) antibody and anti-human GnRH antibody, alternating sections were made. Next, the samples were incubated with PBS containing 0.25% Triton X-100 and 0.25% bovine serum albumin (BSA) (PBS-TX-BSA) for 1 h at 4°C.

The sections were then incubated for 24 h at 4°C with different primary antibodies diluted in PBS-TX-BSA (**Table 2**). After washing with PBS, the samples were incubated for 5 h at 4°C with the respective secondary antibodies diluted in PBS-TX-BSA (**Table 2**). The slides were washed with PBS and cover-slipped with fluorescent mounting medium (#S3023, Dako). The stained tissues were analyzed with a TCS SP8 DMI laser confocal scanning microscope (Leica Microsystems, Germany) equipped with appropriate wavelength-filter configuration settings and a transmitted light detector (BioMarker Ltd, Hungary). Image processing was performed by LasX software (Leica Microsystems, Germany). In the negative control experiments, the sections were only incubated with the secondary antibody and yielded no signal (not shown). The specificity of the anti-ly-GnRH/CRZ antibody (preadsorption with hemocyanin from giant keyhole limpet (KLH), preadsorption with synthetic peptide) was determined previously (49). In some cases, the anti-human GnRH antibody was preadsorbed with 10 µM synthetic human GnRH peptide (#PEP-10933, Thermo Fisher Scientific) overnight at 4°C and applied to the sections resulted in the complete abolishment of the staining (not shown). Given the lack of commercially available proteins, preadsorption control was not performed for the anti-human CYP19A and anti-human nPR antibodies reflecting the methods in the relevant previous papers (8, 10–12, 50).

**Table 2:** Antibodies applied during the immunohistochemical and Western blot procedures.

Antibody	Producer	Dilution
**Primary antibodies**		
rabbit anti-ly-GnRH/CRZ	#AS203-2, EZbiolab	1:1000
rabbit anti-human GnRH	#G8294, Sigma-Aldrich	1:500
mouse anti-human nPR	#MA1-412, Thermo Fischer Scientific	1:200
rabbit anti-human CYP19A	#SAB4500606, Merck	1:500
**Secondary antibodies**		
donkey anti-mouse IgG conjugated with NorthernLights^TM^ NL557	#NL007, R&D System	1:200
donkey anti-rabbit IgG conjugated with NorthernLights^TM^ NL557	#NL004, R&D System	1:200

Given the lack of the relevant genes in mollusks and that the relevant immunogens show no/little homology with non-homologous L. stagnalis proteins (**Supplementary Information**), from the molluscan viewpoint, the three vertebrate antibodies are referred to as non-specific antibodies. The anti-ly-GnRH/CRZ antibody is referred to as a specific antibody.

### Western blot analysis and mass spectrometry identification

The whole CNS was dissected from the animals (n=12). The CNSs were pooled and extracted following our previously published protocol (51). Briefly, the sample was homogenized in 1 mL lysis buffer (1 M TRIS (pH=6.8), 2% SDS, 10% glycerol, 1% mercaptoethanol, 10% protease inhibitory cocktail (#P2714-1BTL, Sigma-Aldrich)) with a Dounce homogenizer and then further extracted with ultra-sonication. After centrifugation (16,000×g for 5 min at 4°C), the supernatant was placed in a new tube. 160 µL of the supernatant was diluted with 5x sample buffer (0.1 M TRIS (pH=6.8), 0.5 M DTT, 2% SDS, 10% glycerol, 1 mM EDTA, 0.1% bromophenol blue) and denatured for 5 min at 95°C.

For the WB analysis, 5 µL samples were run three times on a 10% SDS-PAGE, each with the ProSieve™ QuadColor™ Protein Marker (#00193837, Lonza). In the parallel SDS-PAGE, 20 µL samples were run three times for the MS analysis. The gel then was blotted for two hours onto a nitrocellulose membrane (#GE10600002, Sigma-Aldrich) following the standard semi-dry blotting protocol, while the parallel gel was stained with Coomassie brilliant blue following the standard protocol. The membrane was incubated for 3 h with a blocking solution (10% non-fat dry milk powder and 6% BSA in Tris-buffered saline (TBS; 20 mM Tris, 150 mM NaCl, pH=7.6)), then washed thoroughly (3 x 5 min in TBS with 1% Tween-20 (TBST), then 3 x 5 min in TBS). The membrane was then divided into three distinct pieces and incubated overnight at 4°C with the respective primary antibodies (**Table 2**), each diluted 1:1000 in TBST-6% BSA. After washing (3 x 5 min in TBST, then 3 x 5 min in TBS), the membranes were incubated for 3 h at room temperature with a goat anti-rabbit IgG secondary antibody conjugated with HRP (#172-1019, BioRad) or with a donkey anti-mouse IgG secondary antibody conjugated with HRP (#170-6516, BioRad) – each diluted 1:3000 in TBST-2% non-fat dry milk powder. After washing (3 x 5 min in TBST, 3 x 5 min in TBS), the chemiluminescence was visualized with Western Blotting Luminol Reagent (#sc-2048, Santa Cruz Biotechnology) on an Azure 300 (AZI300-01) system, resulting in digital images. In the negative control experiments, the samples were only probed with the secondary antibody and yielded no bands (not shown). In some cases, the anti-human GnRH antibody was preadsorbed with 10 μM of synthetic human GnRH peptide overnight at 4°C. This resulted in no bands (not shown). Similarly to the IHC method, preadsorption control was not performed for the anti-human CYP19A and anti-human nPR antibodies reflecting the methods in the relevant previous papers (10, 11, 50). Method optimization for Western blotting with the anti-human GnRH antibody to be able to investigate the possible marking at ~1.3 kDa is presented in the **Supplementary Information**.

For MS identification, the respective bands were excised from the parallel SDS-PAGE gel with a razorblade and placed in Eppendorf tubes. After de-staining and cleaning, the in-gel digestion was performed with Trypsin/Lys-C Mix (#V5072, Promega) based on the supplier technical bulletin (fast digestion protocol). Peptides were cleaned on 3 mL SPE tubes (HLB 60 mg; Waters, Milford, USA), and the eluted samples were concentrated with an Eppendorf Concentrator plus system (Eppendorf, Hamburg, Germany). The concentrated peptides were resolved in 100 µL water containing 0.1% formic acid. The proteomics analysis was performed with Bruker EASY-nLC equipment coupled with a nano-ESI MS instrument (Bruker Maxis 4G UHR-QTOF). Three μL aliquots of the samples were injected and separated on a home-made C18 analytical column (3 μm, 75 μm x 150 mm) using a gradient elution at a flow rate of 250 nL min^-1^. Eluents had the following composition: A (aqueous formic acid solution: 0.1%) and B (acetonitrile/formic acid: v/v 99.9/0.1%). The scanning range was 250-2800 m/z. The flow of nebulizer gas was 4 L min^-1^ on 0.6 bar and the temperature was set at 200°C. The capillary voltage was 4.5 kV, and the top 15 peptides were fragmented with the CID fragmentation cell. For protein identification, data were processed with Data Analysis 3.4 software. The identification was carried out with a Mascot server V2.4.1 and PEAKS Studio Xpro software on the Swiss-Prot database. Searching parameters were set to allow one missed cleavage site, accepting 50 ppm mass error at the MS1 and 0.3 Da at the MS2 mode.

## Results

### Uptake of radioactive steroids

**Figure 1:**
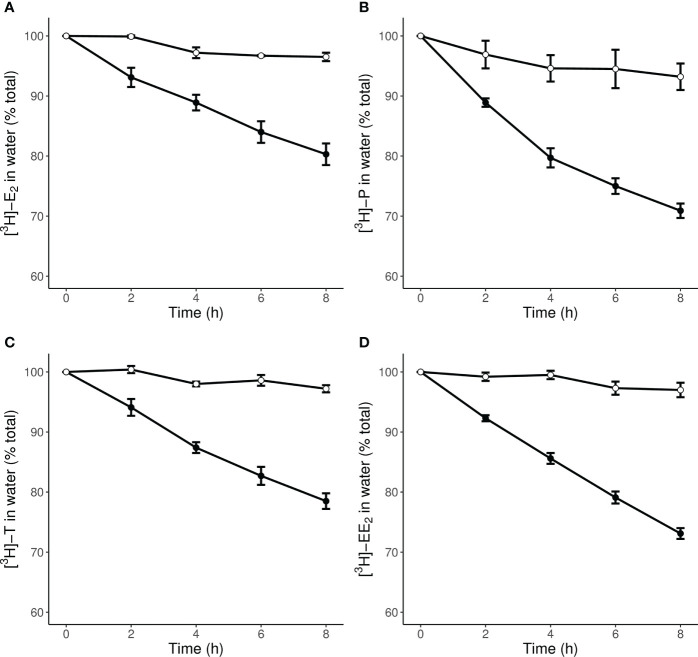
Radioactive steroid removal from water by *L. stagnalis* during an 8 h exposure to a concentration of 2.6 µCi L^-1^ [^3^H]-E_2_ **(A)**, 2.7 µCi L^-1^ [^3^H]-P **(B)**, 3.5 µCi L^-1^ [^3^H]-T **(C)**, and 2.7 µCi L^-1^ [^3^H]-EE_2_ **(D)**. Exposure (●) and sorption control (○) data are presented as mean % total ± S.E.M (n=5 exposure vessels with 2 animals each and n=3 control vessels).

**Figure 1** shows the disappearance of radioactive E_2_, P, T, and EE_2_ from water in five vessels containing two live snails each. There was a marked decrease in radioactivity in all the vessels containing snails with the uptake rate of P being marginally fastest. The amounts of radioactivity remaining in the water after 8 h was ~80% for E_2_, ~70% for P, ~78% for T, and ~75% for EE_2_ treatments. In the first 1.5 h, the calculated clearance rates ranged from 3.7 mL animal^-1^ h^-1^ for E_2_ to 5.6 mL animal^-1^ h^-1^ for P (**Table 3**).

**Table 3:** Clearance rates and associated data.

		Linear Curve(f=y_0_+a*x), constants		Calculated from decay curves:	
Experiment	Removal at end of exposure period (%)	Constant y_o_	Constant a	%removed after first 1.5 h	Clearance rate (mL animal^-1^ h^-1^)
E_2_	19.7	5714.6	-140.3	3.7	3.7
EE_2_	26.7	5857.9	-195.9	5.0	5.0
T	21.5	7686.5	-210.4	4.1	4.1
P	29.1	5864.0	-217.0	5.6	5.6

### Composition of radioactive steroids in snail tissues before and after depuration

**Figure 2:**
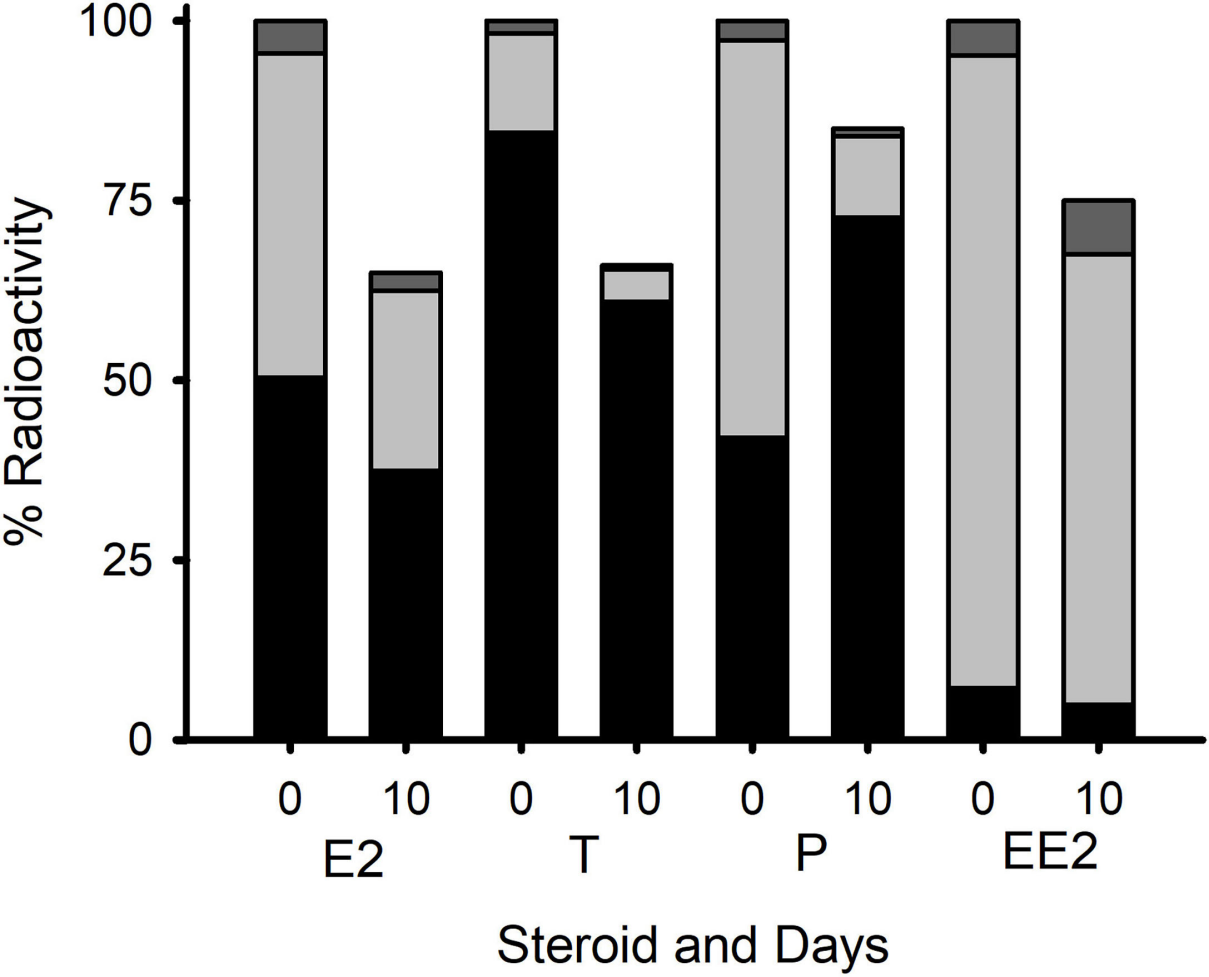
Radioactive residues of four steroids from *L. stagnalis* (whole tissue extracts) on day 0 (i.e. directly after the 8 h of exposure) and day 10 (i.e. after depuration) with day 0 being treated as 100%. The proportions of ester (black), free (light grey), and water-soluble (dark gray) that were found in the extracts on days 0 and 10 are also shown. Data are presented as mean percentage of the total radioactivity in each extract (n=5 for each time point, except for P, where n=4 on day10).

Extraction and phase separation of the metabolites in the snail tissues showed the presence of a high proportion of radioactivity (40, 50 and 85%) for P, E_2_, and T, respectively, in the lipid-rich heptane fraction where esterified steroids would be expected to accumulate (**Figure 2**). The proportion of EE_2_ that had been potentially esterified was, as expected (18), much lower (8%). Most of the rest of the radioactivity was recovered in the 80% ethanol fraction which contains free steroids. Only a small proportion of radioactivity was found in the water-soluble fraction. After ten days of depuration, approximately 70% of the radioactivity was still present in the animals (**Figure 2**). The proportion of steroids in the form of ester (i.e. in the heptane fraction v. the 80% ethanol fraction) was higher for all steroids except EE_2_ - implying the preferential depuration of the free steroids. Only preliminary characterization of the steroids that were present in the ester fraction (following saponification to release the fatty acid moieties) was carried out. This is shown in **Supplementary Figure 3**. There was evidence that most of the E_2_ radioactivity (but not all) eluted in the expected elution position of standard E_2_. The T radioactivity showed two major peaks, one of which eluted with T standard, the other being consistent with the elution position of 5α-DHT. The P radioactivity eluted as two peaks that were consistent with the 5α-reduced steroids identified in the study on the blue mussel *Mytilus edulis* (17). However, it must be noted that these identifications are only tentative. There was insufficient activity in the EE_2_ samples to obtain a meaningful separation.

### Anti-human CYP19A and anti-human nPR antibodies yield a positive signal in the CNS

**Figure 3:**
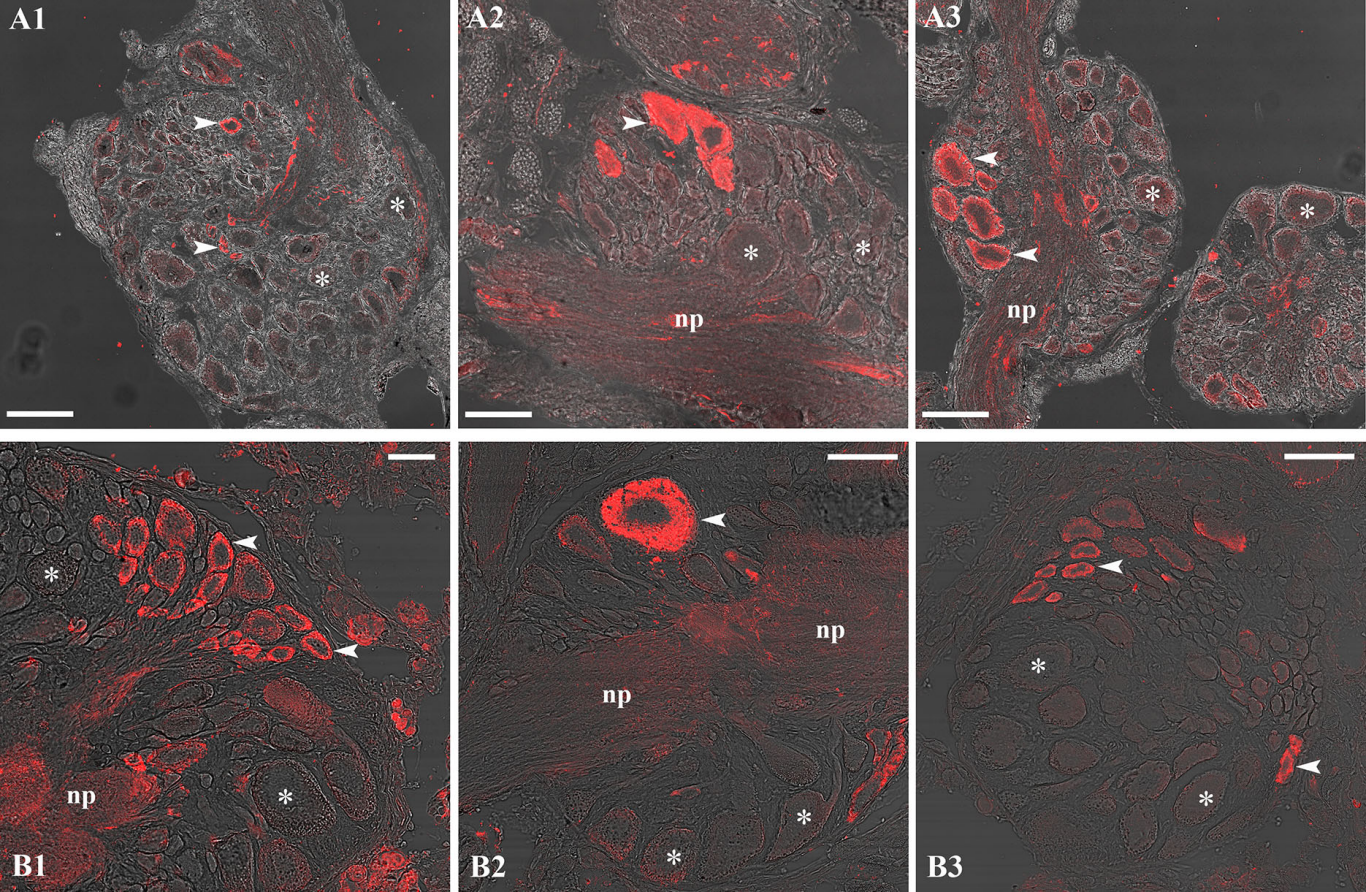
Representative CYP19A (A1-A3) and nPR (B1-B3) immunolabeling (red) in the CNS. The anti-human CYP19A antibody yielded a positive signal, for example, in the right cerebral **(A1)**, right pleural **(A2)**, and right parietal **(A3)** ganglia, while the anti-human nPR antibody gave immunoreactivity, for example, in the left pedal **(B1)** and visceral **(B2, B3)** ganglia. Arrowhead: labeled neuron(s); asterisk: unlabeled neuron(s); np: neuropil. Scale=100 µm **(A1, A3, B2, B3)** and 50 µm **(A2, B1)**.

Despite the lack of CYP19A and nPR genes in *L. stagnalis* (**Supplementary Table 2**), our IHC investigations with the anti-human CYP19A and anti-human nPR antibodies yielded a positive signal in the CNS (**Figure 3**). CYP19A-immunopositive neurons were detected in the paired cerebral (CG) (**Figure 3A1**), paired pleural (PlG) (**Figure 3A2**), paired parietal (PaG) (**Figure 3A3**), unpaired visceral (VG) (**Figure 3A3**), and paired pedal (PeG) (not shown) ganglia. Neurons labeled by the anti-nPR antibody were observed in the PeG (**Figure 3B1**), VG (**Figures 3B2, B3**), and PaG (not shown).

### Comparison of the specific anti-ly-GnRH/CRZ antibody and non-specific anti-human GnRH antibody

**Figure 4:**
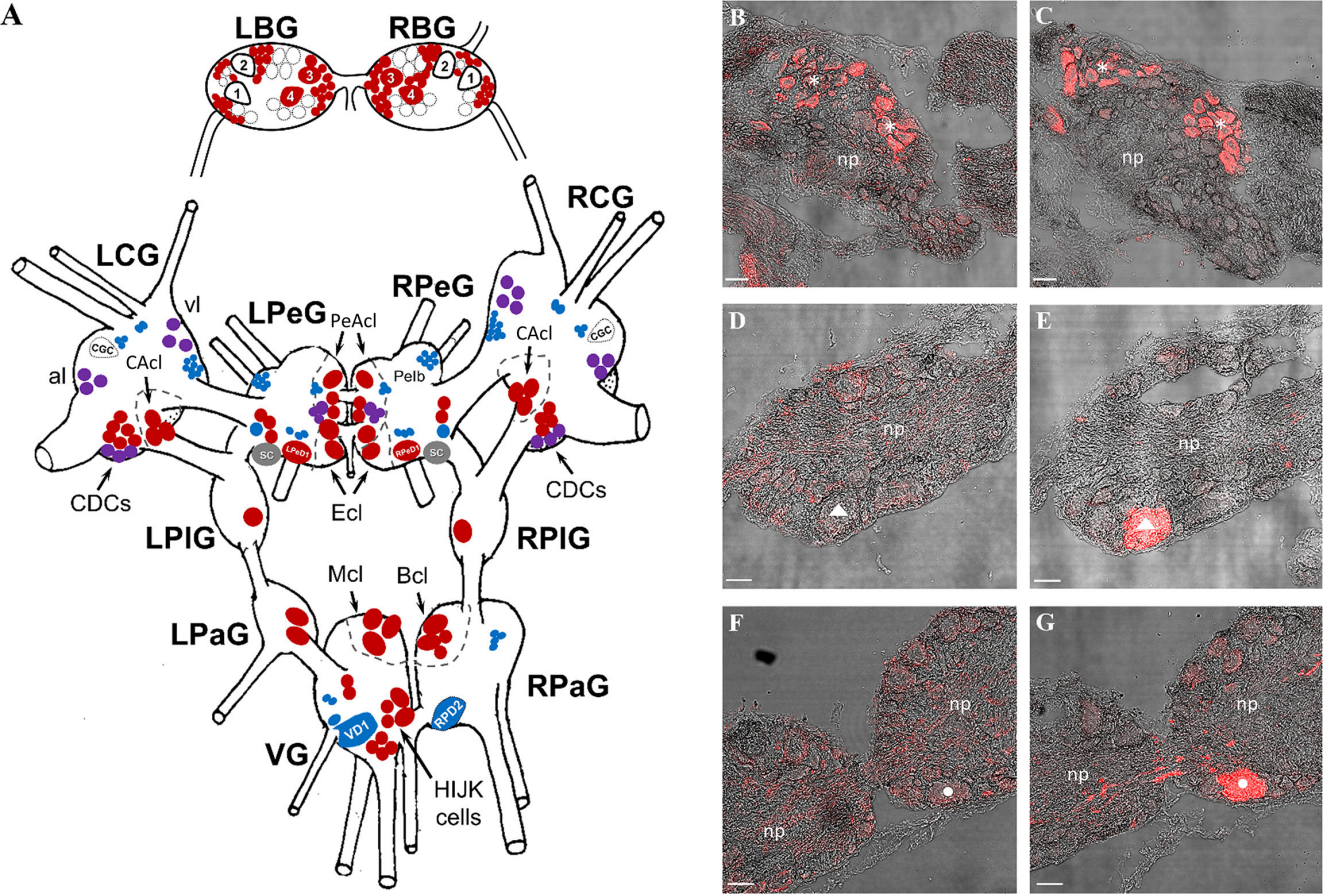
Immunolabeling by the specific anti-ly-GnRH/CRZ antibody [the antigen was a synthetic undecapeptide [CNYHFSNGWYA-amide] corresponding to ly-GnRH/CRZ (49, 52–54)] and/or the non-specific anti-human GnRH antibody (the antigen was a synthetic decapeptide [pQHWSYGLRPG-amide] corresponding to human GnRH) in the CNS. **(A)** Schematic CNS map (dorsal view) showing the distribution of neurons labeled in the left and right buccal (BG), cerebral (CG), pedal (PeG), pleural (PlG), parietal (PaG), and visceral (VG) ganglia. Neurons labeled by the specific, the non-specific, and both antibodies are indicated with red, blue, and purple symbols, respectively. Some unlabeled but identified large neurons (e.g., CGC) are included for providing reference points for anatomical orientation. **(B-G)** Representative micrographs made by alternating sectioning showing the comparative immunolabeling (red). In the right CG **(B, C)**, both specific **(B)** and non-specific **(C)** antibodies yielded a signal, in part the same neurons were labeled (indicated by asterisks). The giant VD1 neuron (marked by a triangle) of the VG **(D, E)** and the giant RPD2 neuron (marked by a circle) of the right PaG **(F, G)** were labeled only by the non-specific antibody **(E, G)**. Scale=50 µm. B1-B4, identified buccal feeding motoneurons; CGC, cerebral giant cell; CDCs, caudo-dorsal cells; al, anterior lobe; vl, ventral lobe; LPeD1, left pedal dorsal 1 neuron; RPeD1, right pedal dorsal 1 neuron; CAcl, cerebral A-cluster; PeAcl, pedal A-cluster; Ecl, E-cluster; SC, statocyst; Mcl, M-cluster; Bcl, B-cluster; VD1, visceral dorsal 1 neuron; RPD2, right parietal dorsal 2 neuron; np, neuropil.

Both specific and non-specific antibodies yielded a positive signal in the CNS (**Figure 4**), however, with a highly different distribution: there were neurons/neuron clusters labeled by both antibodies, while other cells were labeled only by the specific snail or the non-specific human antibody (**Figure 4A**).

In the BG, only the specific antibody yielded an immunopositive signal (e.g., B3 and B4 motoneurons). In the CG, a small subset of caudo-dorsal cells (CDCs) as well as small-sized neurons in the metacerebrum located close to the ventral lobe (vl) and in the anterior lobe (al) were detected by both antibodies (**Figures 4B, C**). A group of medium-sized neurons in the A-cluster (CAcl) and the large majority of CDCs were labeled with the specific antibody. Furthermore, some dispersed, small-sized (unidentified) neurons were detected by the human antibody.

In the PeG, several small- and medium-sized neurons in the A- (PeAcl) and E (Ecl) –clusters, some dispersed (unidentified) neurons, as well as the left serotonergic giant cell (LPeD1) and the right dopaminergic giant neuron (RPeD1) could be labeled by the specific antibody. The human antibody yielded immunoreactivity in pedal lb (Pe lb) clusters as well as in some small (unidentified) neurons around the LPeD1 and RPeD1.

The PlG and the left PaG contained only (unidentified) cells labeled by the specific antibody. Neurons in the right RPaG and VG ganglia were labeled by only the specific (e.g., B-cluster [Bcl], and M-cluster [Mcl]) or only the non-specific (e.g., visceral dorsal one [VD1] – **Figure 4E**; right parietal dorsal two [RPD2] – **Figure 4G**) antibody.

### Identification of proteins labeled by the human antibodies

**Figure 5:**
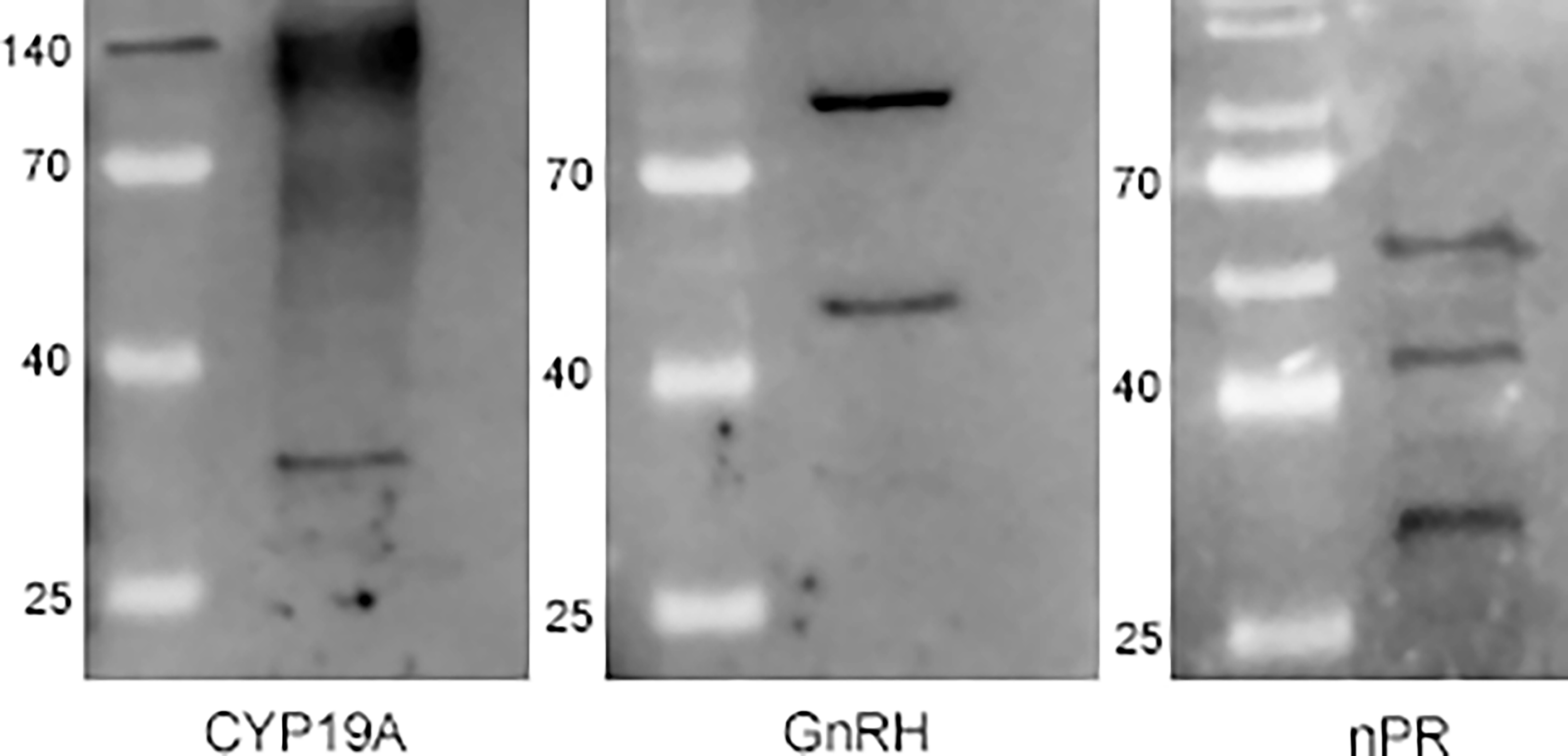
Western blot analysis of the CNS homogenate with the anti-human CYP19A, anti-human GnRH, and anti-human nPR antibodies. The anti-CYP19A antibody yielded one discrete (~30 kDa) and two amorphous (~65 kDa and ~140 kDa) bands, moreover, it marked the 140 kDa marker protein. The anti-GnRH antibody gave two discrete bands (~50 kDa and ~100 kDa), while the anti-nPR antibody resulted in three discrete bands (~30 kDa, ~50 kDa, and ~60 kDa).

To determine which molecules were labeled by the human antibodies, we performed PAGE and WB analysis on the CNS homogenate (**Figure 5**). The anti-human CYP19A antibody yielded one discrete (~30 kDa) and two amorphous (~65 kDa and ~140 kDa) bands. Interestingly, it also marked the 140 kDa marker protein. Instead of the expected band at 1.3 kDa [corresponding to the known mass value of the active ly-GnRH/CRZ peptide (52)], the anti-human GnRH antibody gave two discrete bands with much higher mass value (~50 kDa and ~100 kDa). The anti-human nPR antibody resulted in three discrete bands (~30 kDa, ~50 kDa, and ~60 kDa). The ~30 kDa band was marked by both the anti-human CYP19A and anti-human nPR antibodies, while the ~50 kDa band was marked by both anti-human GnRH and anti-human nPR antibodies.

The subsequent MS identification gave no significant match between the fragments obtained from the bands and the three human protein sequences (or their *in silico* predicted fragments). The proteins that were positively identified in the bands are presented in **Table 4**. However, there was no evidence that any of these proteins were the ones that cross-reacted with the relevant antibodies.

**Table 4:** List of the identified proteins corresponding to excised bands from PAGE by LC-MS analysis.

Sample	Hits
CYP19A: ~30 kDa	Glyceraldehyde-3-phosphate dehydrogenase
CYP19A: ~65 kDa	no hits
CYP19A: ~140 kDa	no hits
Marker protein: ~140 kDa	Bifunctional glutamine synthetase adenylyltransferase/adenylyl-removing enzyme
GnRH: ~50 kDa	Retrograde protein of 51 kDa, tubulin
GnRH: ~110 kDa	Paramyosin
nPR: ~30 kDa	Glyceraldehyde-3-phosphate dehydrogenase
nPR: ~50 kDa	Retrograde protein of 51 kDa, tubulin
nPR: ~60 kDa	no hits

## Discussion

Although vertebrate sex steroids are present in molluscan tissues, it has been argued that they are so readily absorbed from the environment and can be stored for such a long time, that their presence is very poor evidence of endogenous synthesis [reviewed by (4)]. To provide further evidence for this, we exposed *L. stagnalis* to radiolabeled sex steroids and investigated their uptake, metabolism, and depuration. We confirmed that snails do indeed, like many other mollusks, absorb vertebrate steroids. Compared to *M. edulis* (15–18), the rate was approximately ten times slower on a per animal basis. On a gram for gram basis, however, the difference was *c*. four times slower [e.g., the clearance rate for E_2_ was previously demonstrated to be c. 40 mL animal^-1^ h^-1^ for a single mussel (average 3 g wet weight of tissue) (15); for *L. stagnalis* (average 1 g wet weight of tissue), the clearance rate of E_2_ was 3.7 mL animal^-1^ h^-1^]. The fact that mussels are filter feeders and continuously pump water through their gills probably accounts for their overall faster rate of absorption. In mussels, not only was the rate of uptake faster, but also the pattern of disappearance of the steroids from the water fitted hyperbolic or exponential decay functions. In *L. stagnalis*, in contrast, the pattern was linear, except for perhaps P. However, this is probably a result of snail experiments being shorter (8h v. 24h or 48h) and the rate of uptake being slower. Little is known about the mechanism of uptake, although for water-living mollusks it is probably by passive diffusion across the gills. However, sex steroids have been shown to be detected in land-living snails as well (55–57), it is likely that they can also enter the animal by ingestion.

We have confirmed not only that *L. stagnalis* snails can absorb the natural steroids E_2_, T and P from the water, but also that they are able to esterify them. Just as in mussels (18), EE_2_ appeared to be the least easily esterified steroid. Since P has no hydroxyl groups by which it can be esterified, the presence of radioactivity in the ester fraction implied that this steroid had to have been metabolized following uptake. The most likely reactions [based on the steroids previously identified in mussels (17, 25)] are 5α-reduction of the A ring and conversion of the oxo-groups on carbons 3 and 20 to 3β- and 20β-hydroxyl groups, respectively.

Finally, we showed that the steroid radioactivity that could be recovered from the snail tissues was depurated relatively slowly (only *c*. 30% loss over ten days) – implying a half-life of at least two weeks for all four steroids. This is consistent with studies on mussels that showed an initial rapid loss of free steroid and then a very slow loss of the esterified steroid.

As mentioned in the Introduction section, another key line of evidence that, on the surface, appears to support similarities between the molluscan and the vertebrate endocrine systems is that antibodies raised against human proteins that are associated with sex steroid synthesis or receptor-mediation bind to cells in molluscan tissues. Such positive staining with anti-human CYP19A (i.e. aromatase) antibodies (8, 10–12) has been claimed as evidence for the presence of this enzyme in these animals; despite the aromatase gene not being found in the genome of any species outside chordates (2, 3, 36, 58–61). Our own neuronal transcriptome analysis of *L. stagnalis* also failed to reveal CYP19A (**Supplementary Table 2**) (37), yet, like the other studies, we also found positive staining with an anti-human CYP19A. To add complexity to this puzzle, several studies have reported the presence of ‘aromatase activity’ in mollusks (12, 55, 62–66). However, such evidence was mainly obtained indirectly with a non-specific assay [already reviewed by (2, 3)]. In only one case (66) was endogenously-derived estrogen production specifically demonstrated, but the yields were extremely low (<0.1%), suggesting it was due to the now well-recognized phenomenon of enzyme promiscuity (67, 68) (i.e. one or other CYP enzyme having a weak affinity for T or androstenedione that is partially able to catalyze their conversion into estrogens), rather than to the possibility that CYP19A has somehow been missed in numerous previous investigations of molluscan genomes.

We further chose to investigate whether an anti-human nPR antibody gave a signal in the CNS. To the best of our knowledge, the only previous study to look at the immunostaining of an antibody to the nPR in a mollusk was on the common octopus (*Octopus vulgaris*). Like ours, this study also yielded positive staining (50). As with CYP19A, the nPR gene is not present in mollusks (2, 3, 36, 58, 61), the *L. stagnalis* transcriptome data also confirming its absence (**Supplementary Table 2**) (37). This again calls into question the meaningfulness of positive immunostaining with certain vertebrate antibodies for identifying or localizing specific proteins in molluscan tissues.

To study these enigmatic findings further, we compared the immunohistochemical staining of a specific snail antibody with that of a non-specific vertebrate one for ly-GnRH/CRZ neuropeptide in the CNS. When GnRH/CRZ was first described in *O. vulgaris*, the peptide was named ‘invertebrate GnRH’ based on the sequence similarity to human GnRH and its effects observed in the reproductive system (69). Since then we know that these peptides are multifunctional (i.e. they do not have a specific reproductive function as seen in vertebrates) and should most likely be called CRZs (49, 52–54, 70–74). Because of the GnRH term, some previous papers have investigated invertebrate GnRH/CRZ peptides with antibodies raised against vertebrate (tunicate, mammalian, chicken, fish) GnRHs (75–79). Since these investigations yielded a positive signal in the CNS, we expected immunostaining in *L. stagnalis* with the non-specific vertebrate antibody. Although it did give a signal in the CNS, it could not label several genuine ly-GnRH/CRZ-immunopositive cells identified previously (49), but labeled several non-invGnRH/CRZ-containing neurons. This, on the one hand, confirms the need for using appropriate antibodies in order to prevent false conclusions (e.g., inferred functions from the distribution) and, on the other hand, highlights that wrong naming schemes (i.e. nominative determinism) in molluscan research can also contribute to using inappropriate antibodies. To further characterize the proteins that were being labeled by the vertebrate antibodies, we performed PAGE, WB, and MS analyses on a homogenate derived from the CNS. Although WB analyses have previously been performed, subsequent MS identification studies had not been done before. A WB analysis of the ovary and testis homogenates of the mussel *M. galloprovincialis* with an anti-human CYP19A antibody revealed one discrete band at ~60 kDa corresponding to the band of rat CYP19A (10, 11). In contrast, our analysis with an anti-human CYP19A antibody yielded one discrete (~30 kDa) and two amorphous (~65 kDa and ~140 kDa) bands. The antibody also labeled the protein used as the 140 kDa marker. A previous WB investigation of the ovary of *O. vulgaris* with an anti-chicken nPR antibody revealed one discrete band at ~70 kDa (50). In contrast, our analysis with the anti-human nPR antibody yielded three discrete bands (~30 kDa, ~50 kDa, and ~60 kDa).

Since there is a slight sequence similarity between ly-GnRH/CRZ and human GnRH, we did not exclude the possibility that the non-specific antibody can in some cases recognize ly-GnRH/CRZ neuropeptide resulting in some immunopositive-neurons that were also marked by the specific snail antibody. However, instead of one expected band at ~1.3 kDa, the anti-human GnRH antibody gave two discrete bands with much higher mass values (~50 kDa and ~100 kDa) clearly indicating a non-specific signal.

The subsequent MS analysis of the bands found no sequences that were homologous to those found in vertebrate CYP19A, nPR, and GnRH. Although the homolog searching with the immunogen of the anti-human nPR in the *L. stagnalis* sequence data revealed three hits (**Supplementary Information**), they showed no significant match with the fragments obtained from the ~50 kDa band. We could also identify some proteins that may be marked by the antibodies, but this needs to be investigated further.

In summary, we showed that snails, like all other mollusks that have previously been studied, can absorb and accumulate vertebrate steroids. This confirms that the presence of vertebrate sex steroids in molluscan tissues is not necessarily evidence of endogenous production. We also show that the three antibodies that we tested all positively stained two or more proteins in the snail, including, in the case of the CYP19 antibody, one of the molecular weight marker proteins. This clearly highlights that immunostaining with antibodies (especially polyclonal ones) against vertebrate proteins is a highly unreliable procedure for identifying or localizing specific proteins in invertebrate tissues. Our results add to a growing body of literature questioning whether natural selection in mollusks would have favored the evolution of a steroid-based endocrine system. Besides sex steroids (progestogens, androgens, and estrogens), glucocorticoids and mineralocorticoids are the main vertebrate steroid hormones. To the best of our knowledge, there is no evidence that mollusks have 11-hydroxylase and 21-hydroxylase activity (i.e. no corticosterone and cortisol production) or aldosterone synthase activity (i.e. no aldosterone production). Furthermore, the protein homologues of the enzymes that catalyze these reactions (CYP21, CYP11B1, and CYP11B2) are not present in molluscan genomes (80, 81). Also, there is no evidence that mollusks have corticoid receptors (NR3C1 and NR3C2) – the NR3C receptor family is vertebrate-specific (58, 61, 82). Instead of vertebrate-like steroids, we would like to highlight the potential role of sterols as hormones in molluscan endocrinology, though not necessarily in relation to reproduction. For example, non-aromatized steroids (epidioxysterols) have been identified in *Aplysia* (83). Moreover, since aromatized long-chained steroids (e.g., geodisterol) have been firmly identified in sponges and cnidarians (84, 85),they may also be present in mollusks. The potential role of sterols is supported by a recent finding that inhibition of 5α-reductase, that is known to use sterols, as well as steroids as substrates, caused marked malformations in shell morphology during the development of two freshwater gastropods (86). It can be speculated that in mollusks, some of those sterols might bind to nuclear receptors from the NR1H or NR1I/J/K families. In our opinion, this is a better direction to search for nuclear receptor-based signaling in lophotrochozoans than to continually carry out research on the basis that their endocrine systems are the same as in humans.

We hope that readers will take away the message that research that is carried out on the unquestioning assumption that mollusks are ‘honorary humans’ constitutes a massive waste of resources.

## Data availability statement

The raw data supporting the conclusions of this article will be made available by the authors, without undue reservation.

## Author contributions

IF: Conceptualization, Investigation, Writing - original draft, Data curation, Visualization, Funding acquisition. TS: Investigation, Writing - review & editing, Data curation, Visualization. BK: Investigation, Writing - review & editing. AT: Investigation, Writing - review & editing. JS: Investigation, Writing - review & editing, Data curation, Funding acquisition. AC: Data curation, Visualization, Writing - review & editing. IK: Investigation, Writing - review & editing, Supervision, Funding acquisition. AS: Conceptualization, Methodology, Writing - review & editing. ZP: Conceptualization, Methodology, Writing - review & editing, Data curation, Visualization, Supervision, Funding acquisition. All authors contributed to the article and approved the submitted version.

## Funding

This work was supported by the National Brain Project (#2017-1.2.1 NKP-2017-00002; ZP); Hungarian Scientific Research Fund (#138039; ZP); Bolyai Foundation (#BO/00646/21/8; ZP); László János Doctoral Scholarship (#498/2021/PTE DOK; IF); New National Excellence Program (ÚNKP-20-3-II-PTE-888; IF) and Cooperative Doctoral Programme for Doctoral Scholarships (KDP-2020-1018493; IF) of the Ministry for Innovation and Technology from the source of the National Research, Development and Innovation Fund; Scholarship for National Young Talents (NTP-NFTÖ-21-B-0212; IF); Defra (#CB0485); Cefas internal funds (Cefas Seedcorn); and Thematic Excellence Program (#TKP2021-EGA-17; JS).

## Acknowledgments

The authors thank Prof. Pei-San Tsai for kindly providing the specific GnRH/CRZ antibody. The research was performed in collaboration with Mass Spectrometry Core Facility at the Szentágothai Research Centre of the University of Pécs.

## Conflict of interest

The authors declare that the research was conducted in the absence of any commercial or financial relationships that could be construed as a potential conflict of interest.

## Publisher’s note

All claims expressed in this article are solely those of the authors and do not necessarily represent those of their affiliated organizations, or those of the publisher, the editors and the reviewers. Any product that may be evaluated in this article, or claim that may be made by its manufacturer, is not guaranteed or endorsed by the publisher.
