# Supplementary material for: Studies on a widely-recognized snail model species (Lymnaea stagnalis) provide further evidence that vertebrate steroids do not have a hormonal role in the reproduction of mollusks
Source: Front Endocrinol (Lausanne). 2022 Sep 8;13:981564. doi: 10.3389/fendo.2022.981564 (PMC9493083; doi:10.3389/fendo.2022.981564)
Supplement: Supplementary file 1 [file DataSheet_1.docx]

**Supplementary information**

**Studies on a widely-recognized snail model species (*Lymnaea stagnalis*) provide further evidence that vertebrate steroids do not have a hormonal role in the reproduction of mollusks.**

^1^István Fodor*^,+^, ^2^Tamar Schwarz^+^, ^3^Bence Kiss, ^3^Antal Tapodi, ^3^János Schmidt, ^4^Alex R. O. Cousins, ^2^Ioanna Katsiadaki, ^2^Alexander P. Scott, and ^1^Zsolt Pirger

^1^Ecophysiological and Environmental Toxicological Research Group, Balaton Limnological Research Institute, Eötvös Loránd Research Network (ELKH), Klebelsberg Kuno u. 3., H-8237 Tihany, Hungary

^2^Centre for Environment, Fisheries and Aquaculture Research (Cefas), Barrack Road, Weymouth, DT4 8UB, United Kingdom

^3^Institute of Biochemistry and Medical Chemistry, Medical School, University of Pécs, 7624, Pécs, Hungary

^4^Centre for Environment, Fisheries and Aquaculture Science, Lowestoft Laboratory, Lowestoft, United Kingdom

*Corresponding author; E-mail: [fodor.istvan@blki.hu](mailto:fodor.istvan@blki.hu) (I. Fodor)

^+^Equally contributed authors

**Supplementary Figure 1.** Simplified (‘delta-5-steroids’ formed from pregnenolone are not shown) vertebrate sex steroidogenesis pathway (modified after ([1](#_ENREF_1))). Six reactions (solid arrows), and the enzymes that catalyze them, appear to be present in mollusks, though some with low activity. CYP11A and CYP19A (marked in italics) have not been found in molluscan genomes so far, this supports the notion that two crucial steps - cholesterol side-chain cleavage and aromatization (dashed arrows) - of the classical vertebrate steroid biosynthetic pathway are absent in mollusks.

**
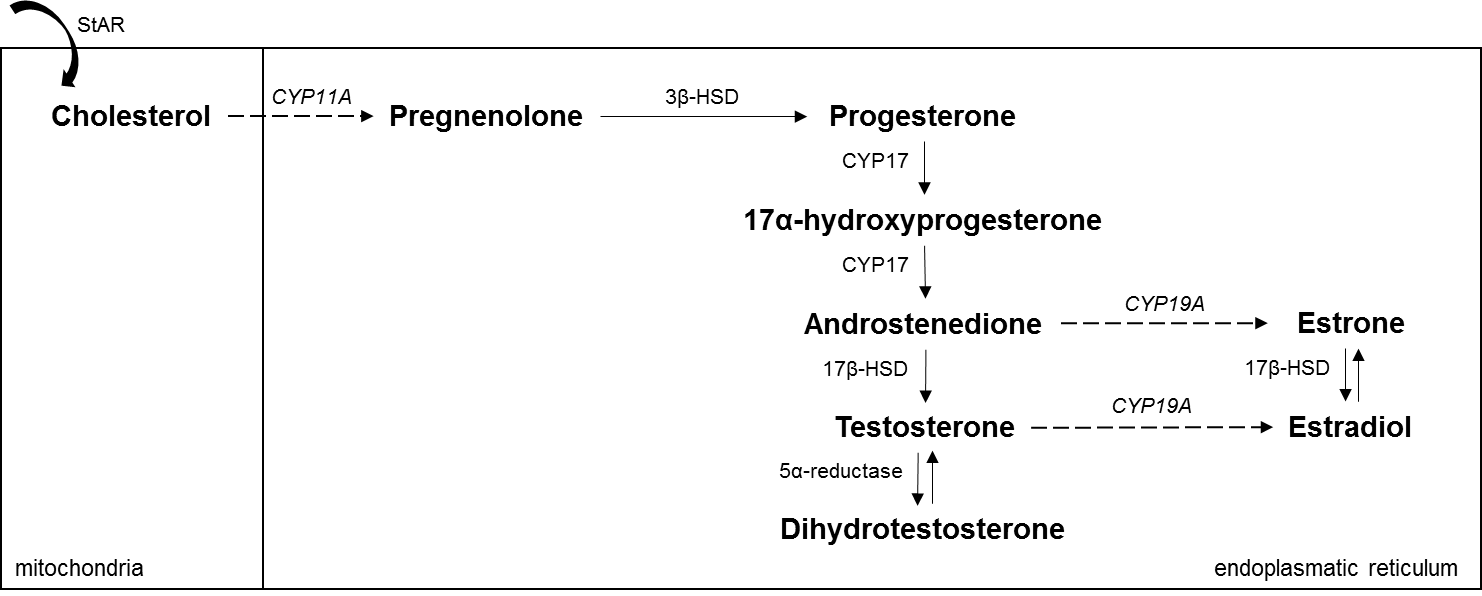
**

**Supplementary Table 1.** Known molluscan homologs of genes involved in receptor-mediation of vertebrate sex steroids (based on ([2-6](#_ENREF_2))). Green color indicates the presence of a homologous sequence in mollusks, while red color represents its absence.

Although a homolog sequence to the vertebrate nuclear estrogen receptor (nER) can be found in mollusks, it is not predicted to bind estrogens (i.e. it is not a functional estrogen receptor and its terminology needs to be revised). The molluscan estrogen receptor homologues display a lower degree of sequence similarity compared with the vertebrate equivalents supporting a hypothesis that there may be different targets (see Supplementary Figure 2).

Neither the gene for nuclear androgen receptor (nAR) nor nuclear progesterone receptor (nPR) can be found in molluscan genomes. Regarding membrane progesterone receptors (mPR), homologs of mPRβ and mPRγ are present in mollusks but their progesterone-binding ability has not been tested and their presence does not necessarily mean the same function as in vertebrates. Importantly, mPRα mediates most mPR functions in vertebrate cells.

Homologous sequences to membrane-associated progesterone receptors, including progesterone membrane receptor component (PGMRC) 1, are also present in mollusks, but the current prevailing view is that they do not actually bind progesterone even in vertebrates.

| **Receptor** | nER | nAR | nPR | mPRα | mPRβ | mPRγ | mPRδ | mPRε | PGMRC1 | PGMRC2 | neudesin | neuferricin |
| --- | --- | --- | --- | --- | --- | --- | --- | --- | --- | --- | --- | --- |
| **Homologs in mollusks** |  |  |  |  |  |  |  |  |  |  |  |  |

**Supplementary Figure 2.** Comparing the protein sequence similarity of the ligand binding domain (cd06949) of *Oncorhynchus mykiss* estrogen receptor (#NP_001117821.1) to vertebrate and molluscan homologues using SeqAPASS ([7](#_ENREF_7)). Molluscan and vertebrate taxa are indicated in the legend.


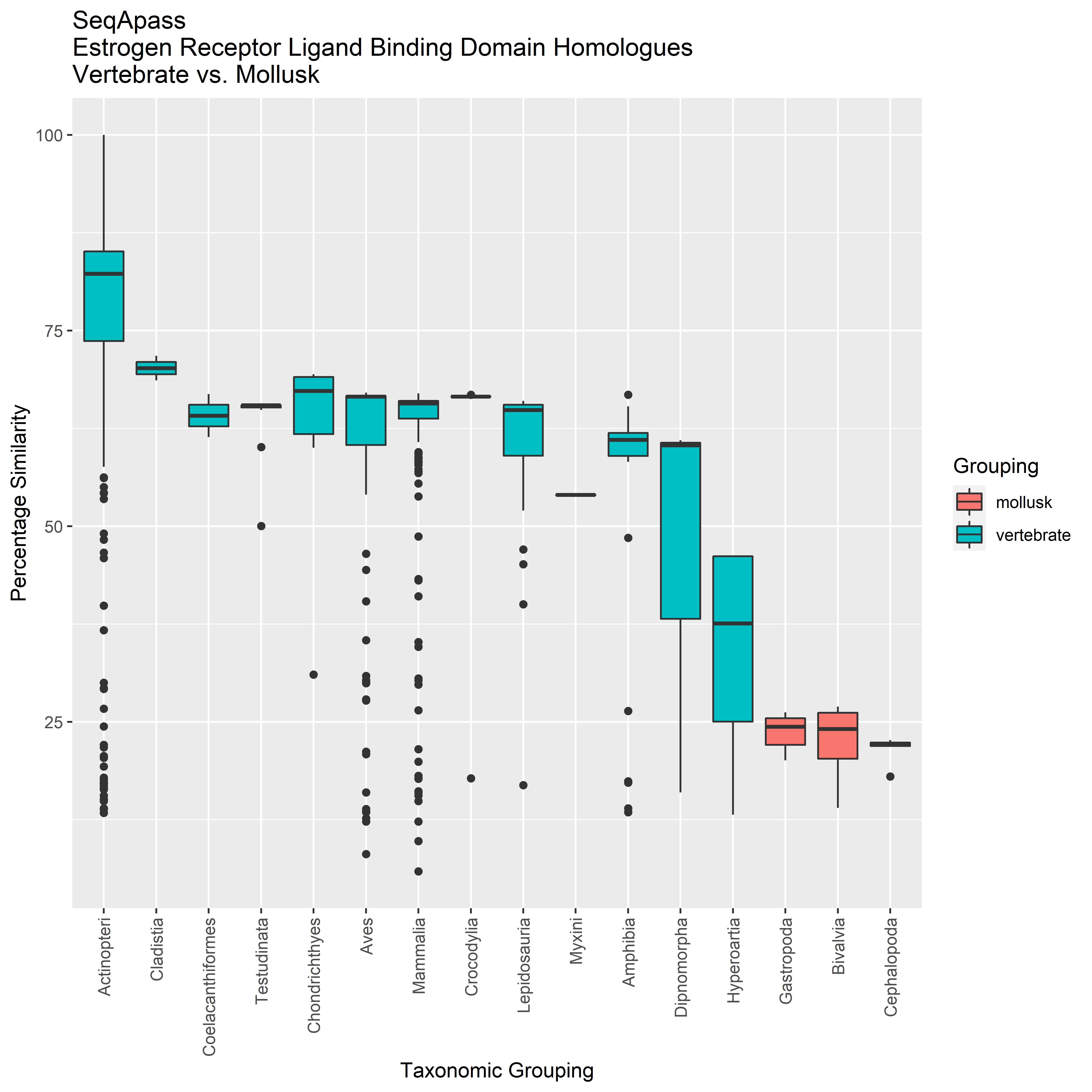


**Supplementary Table 2.** Homologs of genes involved in the vertebrate sex steroidogenesis pathway and receptor-mediation identified in *L. stagnalis* (adapted from ([1](#_ENREF_1))).

| **Vertebrate sex steroidogenesis pathway enzymes and sex steroid receptors** | **Homolog(s) in *L. stagnalis*** |
| --- | --- |
| Steroidogenic acute regulatory protein (StAR) | found |
| Side-chain cleavage enzyme (CYP11A) | not found |
| 3α-hydroxysteroid dehydrogenase (3α-HSD) | not found |
| 3β-hydroxysteroid dehydrogenase (3β-HSD) | found |
| 17α-hydroxylase/17,20-lyase (CYP17) | found |
| 17β-hydroxysteroid dehydrogenase (17β –HSD) | found |
| 5α-reductase | found |
| Aromatase (CYP19A) | not found |
| Nuclear estrogen receptor alpha (nERα/ NR3A1) | found |
| Nuclear progesterone receptor (nPR/NR3C3) | not found |
| Nuclear androgen receptor (nAR/NR3C4) | not found |
| Progesterone receptor membrane component 1 (PGRMC1) | found |
| Steroid binding globulins (SBGs) | not found |

**Supplementary Figure 3.** Characterization of steroids that are present in the ester fraction. Representative HPLC chromatogram of tissue extract after hydrolysis of lipophilic [^3^H]-E_2_ metabolites with E_2_ and E_2_-17 sulphate standard (**A**), [^3^H]-P metabolites with P standard (**B**), [^3^H]-T metabolites with T standard (**C**). Data are presented as radioactivity peaks and elution times of standards (UV, 280 nm).


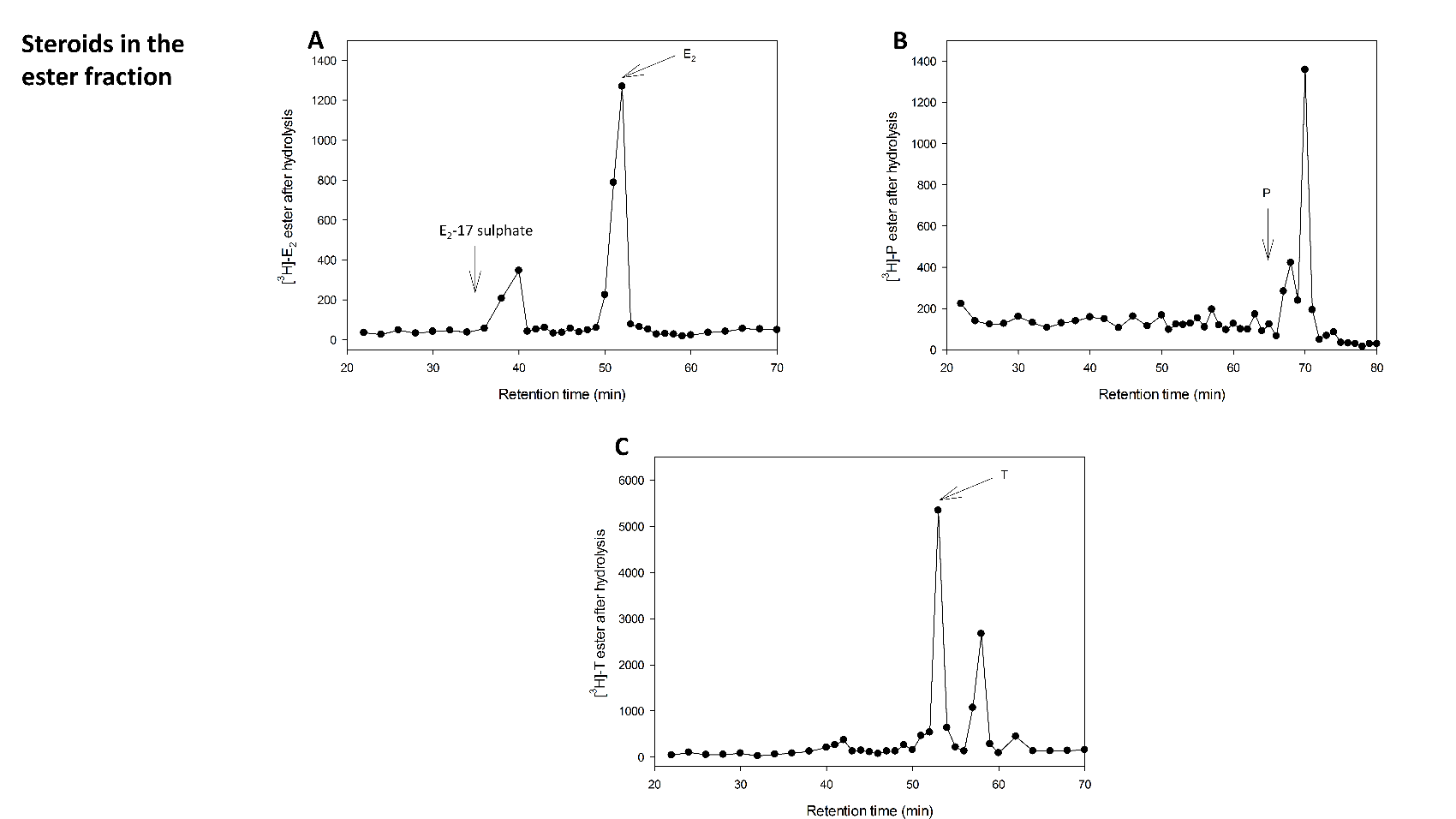


It was found that >79% of the activity elutes in the exact position of standard E_2_ (Fig. S2A). A smaller peak with a retention time of 38-40 min is also present but remains unidentified.

In the case of [^3^H]-P (Fig. S2B), there was no activity at the elution position of the P standard. This is expected as P has no reactive hydroxyl group for esterification to occur, and would therefore require prior metabolism. Two major peaks eluted closely with retention times of 68 and 70 min and corresponding activity of 24 and 76%. These highly non-polar metabolites remain to be identified.

In the case of [^3^H]-T (Fig. S2C), 51% of the activity co-eluted with the T standard and a second major peak with a retention time of 57-58 min accounted for 36% of the total activity. Several minor peaks (42, 52 and 62 min; 4, 5 and 5% respectively) were also present, which, contrary to E_2_, suggests that T is heavily metabolized before undergoing esterification. The putative identification of the T peak and the suspected 5α-DHT (peak 57-58 min; based on T metabolism by mussels and the retention time) should be confirmed with a different chromatographic method (e.g. TLC). The minor peaks are not present in high enough concentrations for further investigation (insufficient product).

It should be noted that all hydrolyzed ester fractions were obtained by pooling heptanes from separations of individuals from before and after depuration (due to low activity), and therefore have no bearing regarding changes in metabolite profile over time. The methodology including the HPLC conditions was the same as described previously ([8](#_ENREF_8)).

**Detailed information about the used primary antibodies**

1) anti-ly-GnRH/CRZ antibody

- Immunogen: CNYHFSNGWYA-amide conjugated to KLH, corresponding to the active ly-GnRH/CRZ peptide (QNYHFSNGWYA-amide)

- Identification of the ly-GnRH/CRZ coding sequence by nucleotide sequencing and verification of the active peptide by mass spectrometry have previously been performed ([9](#_ENREF_9), [10](#_ENREF_10)).

2) anti-human GnRH antibody

- Immunogen: pQHWSYGLRPG-amide without conjugation

- The protein sequence gives no hits using NCBI Blast on *L. stagnalis* or using our unpublished *L. stagnalis* neural transcriptome/whole genome data, i.e. no shared sequence with non-homologous *L. stagnalis* proteins.

3) anti-human CYP19A antibody

- Immunogen range: 221-270 amino acids of human aromatase (NCBI Reference Sequence: NP_001334185.1) without conjugation. Reactivity with many vertebrate taxa.

- Neither the immunogen range nor the entire protein sequence give hits using NCBI Blast on *L. stagnalis* or using our unpublished *L. stagnalis* neural transcriptome/whole genome data, i.e. no shared sequence with non-homologous *L. stagnalis* proteins.

4) anti-human nPR antibody

- Immunogen: nuclear progesterone receptor purified from chick oviduct cytosol (NCBI Reference Sequence: NP_990593.1) without conjugation. Reactivity with many vertebrate taxa. Most of the relevant IHC in human tissues use anti-nPR antibodies generated against small fragments of human nPR or entire chick nPR.

- Using NCBI Blast on *L. stagnalis*, the search with the entire protein sequence yields the previously identified nER homolog (#QIH29348.2; ~43 kDa), retinoid X receptor (#AAW34268.1; ~48 kDa), and retinoic acid receptor (#ADF43963.1; ~52 kDa) with very low homology (~25%) in every case.

**Method optimization for Western blotting with the anti-human GnRH antibody**

The used extraction method previously proved to be suitable for detecting low kDa (e.g., 4-5 kDa) peptides from the CNS of *L. stagnalis* in Western blotting. In order to ensure an appropriate, better resolution separation, we also used 16% PAGE instead of 10%. Compared to Figure 5 in which the 25 kDa marker was the lowest band, this resulted in the separation of the 25, 15, 10, and 4.6 kDa marker bands. We also checked if we could reach a better resolution with a tricine gel system (stacking gel: 4%; intermediate gel: 10%; separating gel: 15%). To optimize the blotting procedure, we also tried a PVDF membrane (pore size: 0.22 μm) instead of the larger pored nitrocellulose membrane (pore size: 0.45 μm) and lower voltage. Representative Supplementary Figure 4 shows that only the ~50 and ~100 kDa bands were marked in these cases as well (5, 10, and 15 µL samples were run for the analysis).


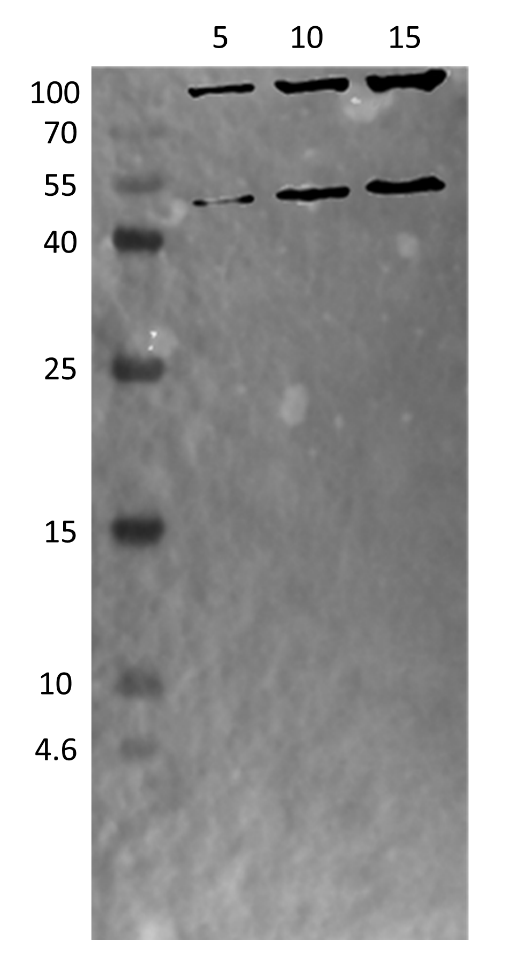


**References**

1. Fodor I, Koene JM, Pirger Z. Neuronal Transcriptome Analysis of a Widely Recognised Molluscan Model Organism Highlights the Absence of Key Proteins Involved in the *de novo* Synthesis and Receptor-Mediation of Sex Steroids in Vertebrates. *Malacologia* (2021) 64(1):69-77. doi: 10.4002/040.064.0103

2. Fodor I, Urban P, Scott AP, Pirger Z. A Critical Evaluation of Some of the Recent So-Called 'Evidence' for the Involvement of Vertebrate-Type Sex Steroids in the Reproduction of Mollusks. *Mol Cell Endocrinol* (2020) 516:110949. doi: 10.1016/j.mce.2020.110949

3. Scott AP. Do Mollusks Use Vertebrate Sex Steroids as Reproductive Hormones? Part I. Critical Appraisal of the Evidence for the Presence, Biosynthesis and Uptake of Steroids. *Steroids* (2012) 77:1450-68. doi: 10.1016/j.steroids.2012.08.009

4. Markov GV, Gutierreze-Mazariegos J, Pitrat D, Billas IML, Bonneton F, Moras D, et al. Origin of an Ancient Hormone/Receptor Couple Revealed by Resurrection of an Ancestral Estrogen. *Sci Adv* (2017) 3:e1601778. doi: 10.1126/sciadv.1601778

5. Ren J, Chung-Davidson YW, Jia L, Li W. Genomic Sequence Analyses of Classical and Non-Classical Lamprey Progesterone Receptor Genes and the Inference of Homologous Gene Evolution in Metazoans. *BMC Evol Biol* (2019) 19(1):136. doi: 10.1186/s12862-019-1463-7

6. Fodor I, Pirger Z. From Dark to Light - an Overview of over 70 Years of Endocrine Disruption Research on Marine Mollusks. *Front Endocrinol* (2022) 13:903575. doi: 10.3389/fendo.2022.903575

7. LaLone CA, Villeneuve DL, Lyons D, Helgen HW, Robinson SL, Swintek JA, et al. Editor's Highlight: Sequence Alignment to Predict across Species Susceptibility (Seqapass): A Web-Based Tool for Addressing the Challenges of Cross-Species Extrapolation of Chemical Toxicity. *Toxicol Sci* (2016) 153(2):228-45. doi: 10.1093/toxsci/kfw119

8. Schwarz TI, Katsiadaki I, Maskrey BH, Scott AP. Mussels (*Mytilus* spp.) Display an Ability for Rapid and High Capacity Uptake of the Vertebrate Steroid, Estradiol-17β from Water. *J Steroid Biochem Mol Biol* (2017) 165(Pt B):407-20. doi: 10.1016/j.jsbmb.2016.08.007

9. Fodor I, Svigruha R, Bozso Z, Toth GK, Osugi T, Yamamoto T, et al. Functional Characterization and Related Evolutionary Implications of Invertebrate Gonadotropin-Releasing Hormone/Corazonin in a Well-Established Model Species. *Sci Rep* (2021) 11(1):10028. doi: 10.1038/s41598-021-89614-5

10. Fodor I, Zrinyi Z, Urban P, Herczeg R, Buki G, Koene JM, et al. Identification, Presence, and Possible Multifunctional Regulatory Role of Invertebrate Gonadotropin-Releasing Hormone/Corazonin Molecule in the Great Pond Snail (*Lymnaea stagnalis*). *Gen Comp Endocrinol* (2020) 299:113621. doi: 10.1016/j.ygcen.2020.113621
